# Clinical Efficacy of Combined Surgical Patient Safety System and the World Health Organization’s Checklists in Surgery: A Nonrandomized Clinical Trial

**DOI:** 10.1001/jamasurg.2020.0989

**Published:** 2020-05-13

**Authors:** Anette Storesund, Arvid Steinar Haugen, Hans Flaatten, Monica W. Nortvedt, Geir Egil Eide, Marja A. Boermeester, Nick Sevdalis, Øystein Tveiten, Ruby Mahesparan, Bjørg Merete Hjallen, Jonas Meling Fevang, Catrine Hjelle Størksen, Heidi Frances Thornhill, Gunnar Helge Sjøen, Solveig Moss Kolseth, Rune Haaverstad, Oda Kristine Sandli, Eirik Søfteland

**Affiliations:** 1 Department of Anesthesia and Intensive Care, Haukeland University Hospital, Bergen, Norway; 2 Department of Clinical Medicine, Faculty of Medicine, University of Bergen, Bergen, Norway; 3 Centre for Evidence-Based Practice, Western Norway University of Applied Sciences, Bergen, Norway; 4 Centre for Clinical Research, Haukeland University Hospital, Bergen, Norway; 5 Department of Global Public Health and Primary Care, University of Bergen, Bergen, Norway; 6 Department of Surgery, Academic Medical Center, Amsterdam, the Netherlands; 7 Center for Implementation Science, Health Service and Population Research Department, King’s College, London, United Kingdom; 8 Department of Neurosurgery, Haukeland University Hospital, Bergen, Norway; 9 Department of Orthopedic Surgery, Haukeland University Hospital, Bergen, Norway; 10 Department of Gynecology and Obstetrics, Haukeland University Hospital, Bergen, Norway; 11 Department of Anesthesiology, Haugesund Hospital, Health Trust Fonna, Haugesund, Norway; 12 Section of Cardiothoracic Surgery, Department of Heart Disease, Haukeland University Hospital, Bergen, Norway; 13 Department of Clinical Science, Faculty of Medicine, University of Bergen, Bergen, Norway; 14 Department of Surgery, Førde Central Hospital, Førde, Norway

## Abstract

**Importance:**

Checklists have been shown to improve patient outcomes in surgery. The intraoperatively used World Health Organization surgical safety checklist (WHO SSC) is now mandatory in many countries. The only evidenced checklist to address preoperative and postoperative care is the Surgical Patient Safety System (SURPASS), which has been found to be effective in improving patient outcomes. To date, the WHO SSC and SURPASS have not been studied jointly within the perioperative pathway.

**Objective:**

To investigate the association of combined use of the preoperative and postoperative SURPASS and the WHO SSC in perioperative care with morbidity, mortality, and length of hospital stay.

**Design, Setting, and Participants:**

In a stepped-wedge cluster nonrandomized clinical trial, the preoperative and postoperative SURPASS checklists were implemented in 3 surgical departments (neurosurgery, orthopedics, and gynecology) in a Norwegian tertiary hospital, serving as their own controls. Three surgical units offered additional parallel controls. Data were collected from November 1, 2012, to March 31, 2015, including surgical procedures without any restrictions to patient age. Data were analyzed from September 25, 2018, to March 29, 2019.

**Interventions:**

Individualized preoperative and postoperative SURPASS checklists were added to the intraoperative WHO SSC.

**Main Outcomes and Measures:**

Primary outcomes were in-hospital complications, emergency reoperations, unplanned 30-day readmissions, and 30-day mortality. The secondary outcome was length of hospital stay (LOS).

**Results:**

In total, 9009 procedures (5601 women [62.2%]; mean [SD] patient age, 51.7 [22.2] years) were included, with 5117 intervention procedures (mean [SD] patient age, 51.8 [22.4] years; 2913 women [56.9%]) compared with 3892 controls (mean [SD] patient age, 51.5 [21.8] years; 2688 women [69.1%]). Parallel control units included 9678 procedures (mean [SD] patient age, 57.4 [22.2] years; 4124 women [42.6%]). In addition to the WHO SSC, adjusted analyses showed that adherence to the preoperative SURPASS checklists was associated with reduced complications (odds ratio [OR], 0.70; 95% CI, 0.50-0.98; *P* = .04) and reoperations (OR, 0.42; 95% CI, 0.23-0.76; *P* = .004). Adherence to the postoperative SURPASS checklists was associated with decreased readmissions (OR, 0.32; 95% CI, 0.16-0.64; *P* = .001). No changes were observed in mortality or LOS. In parallel control units, complications increased (OR, 1.09; 95% CI, 1.01-1.17; *P* = .04), whereas reoperations, readmissions, and mortality remained unchanged.

**Conclusions and Relevance:**

In this nonrandomized clinical trial, adding preoperative and postoperative SURPASS to the WHO SSC was associated with a reduction in the rate of complications, reoperations, and readmissions.

**Trial Registration:**

ClinicalTrials.gov Identifier: NCT01872195

## Key Points

**Question:**

Does patient safety improve when adding the preoperative and postoperative Surgical Patient Safety System checklists to the World Health Organization’s established surgical safety checklist?

**Findings:**

In this stepped-wedge cluster nonrandomized clinical trial with parallel controls that included 9009 surgical procedures, reductions in complications and emergency reoperations occurred when the preoperative Surgical Patient Safety System was added to the surgical safety checklist. The postoperative Surgical Patient Safety System reduced readmissions, whereas overall increased complications were found in the 9678 parallel controls.

**Meaning:**

These findings suggest that joint use of the preoperative and postoperative Surgical Patient Safety System with the intraoperative surgical safety checklist is beneficial for patients.

## Teaser

This nonrandomized clinical trial investigates the association of combining the preoperative and postoperative Surgical Patient Safety System with the World Health Organization surgical safety checklist in perioperative care with morbidity, mortality, and length of hospital stay among patients in 3 surgical departments.

## Introduction

The World Health Organization surgical safety checklist (WHO SSC), now used globally, has been found to reduce complications and mortality,^1,2,3,4,5,6^ although negative findings have also been published.^7,8^ Questions have been raised regarding whether negative results are due to a lack of emphasis on the implementation and local tailoring.^8,9,10^ The WHO SSC is used within the operating room, aiming to improve teamwork, with shared awareness of the safety aspects of surgery.^11^

However, surgical complications often originate before and after operating room activities.^12,13,14^ The comprehensive Surgical Patient Safety System (SURPASS) developed in the Netherlands is the only surgical safety checklist to date to include specific preoperative and postoperative checklists for individual clinicians in addition to team checks in the operating room. Like the WHO SSC, SURPASS has also been found to reduce complications and mortality.^15^ The effect of implementing SURPASS has been replicated only in a smaller study from India,^16^ which found that use of SURPASS alone reduced the rate of postoperative complications in both elective and emergency operations.

To date, whether surgical safety can improve further when combining the intraoperative WHO SSC with the preoperative and postoperative SURPASS remains unknown. This study aimed to evaluate the associations of adding the preoperative and postoperative SURPASS to the intraoperative WHO SSC with surgical complications, all-cause 30-day mortality, and subsequent length of hospital stay (LOS).

## Methods

### Study Design, Setting, and Oversight

**Figure 1.**
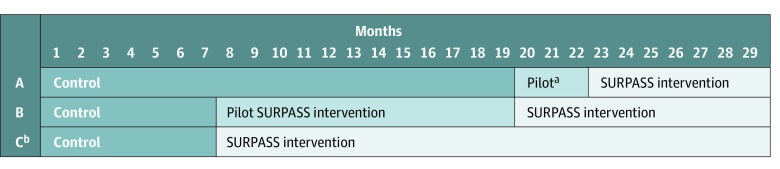
Stepped-Wedge Cluster Nonrandomized Clinical Trial Design Implementation of the individualized preoperative and postoperative Surgical Patient Safety System (SURPASS) checklists in 3 surgical clusters in a tertiary hospital in Western Norway, November 2012 to March 2015 (29 months). A indicates gynecology; B, orthopedics; and C, neurosurgery. ^a^Indicates pilot SURPASS intervention. ^b^Indicates 3-week pilot during June and July 2012.

The trial protocol is given in Supplement 1. A prospective, stepped-wedge cluster nonrandomized clinical trial design^17^ was used. The study implemented the preoperative and postoperative SURPASS checklists in 3 surgical departments in a tertiary hospital in western Norway (Figure 1) in addition to the WHO SSC. The study was approved by the regional ethical research committee, the data privacy units at the Health Trust Førde, and Health Trust Fonna of Norway. After approval, the patients in the intervention departments received written information on the study and their option to refrain from data sharing. The study was exempt from written informed consent. This study followed the extension of the 2010 Consolidated Standards of Reporting Trials (CONSORT) reporting guideline.^18^

At the time of the study (and to date), the WHO SSC is mandatory in Norwegian operating rooms. The study design allowed introduction of the SURPASS to each department sequentially^19^ as opposed to a classic before-after design, wherein the switch from before/control to after/intervention is introduced for all the trial departments at the same time. The original SURPASS^15^ and WHO SSC^20^ intervention trials are classic before-after studies. Our current design allows adjustment for time trends and is advantageous in health care settings with limited resources, involving continuous advancements and change.^19^

Following advice from the WHO checklist implementation guideline, the trial departments were invited to participate based on their management commitment, frontline positive engagement, and high adherence to the WHO SSC.^21^ The SURPASS intervention followed a stepwise introduction in 1 department at a time. The departments each contributed patient data before and after the study intervention and served as their own controls, thus minimizing selection bias. Contamination between study departments and the parallel control departments—caused by information bias due to personnel working in several disciplines, sections, or departments—was avoided: The operating rooms and surgical teams were geographically separate with their own organizational units and specialized personnel (neurosurgery, orthopedic surgery, and gynecology and the parallel control departments of thoracic surgery, general surgery, vascular surgery, gastroenterology, urology, orthopedics, and ear, nose, and throat surgery). Because single surgical procedures were subjects of investigation, it was unlikely that any participant could have been in both control and intervention groups, hence within-department contamination was avoided. Three separate surgical units in different hospitals (a tertiary hospital serving a population of 1.1 million, a community hospital serving a population of 110 000, and a community hospital serving a population of 180 000) constituted additional parallel controls, with the WHO SSC as standard care but without SURPASS.

### Intervention

**Figure 2.**
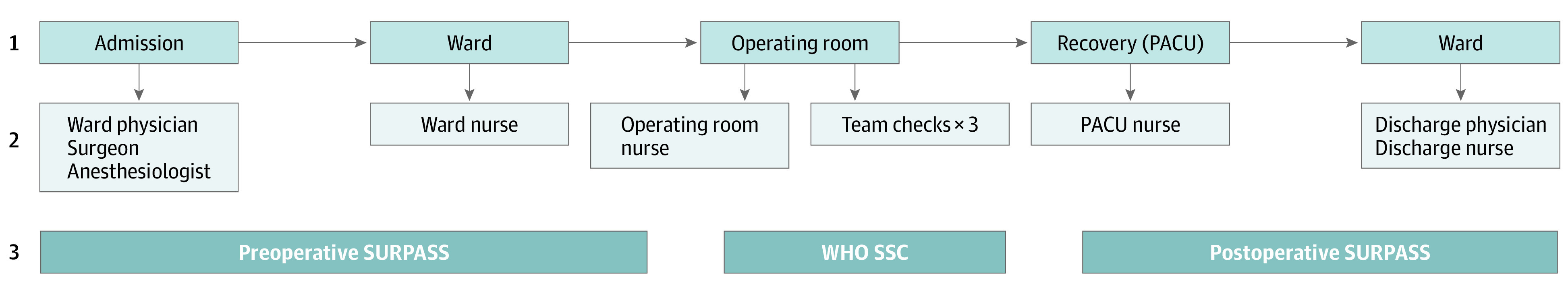
Surgical Checklist Flow 1 indicates surgical pathway; 2, checkpoints for clinicians; and 3, Surgical Patient Safety System (SURPASS) or World Health Organization surgical safety checklist (WHO SSC) applications. PACU indicates postanesthesia care unit.

The original SURPASS system was developed to include known risk factors described in the literature, validated against actual registered adverse events.^14^ The preoperative and postoperative SURPASS checklists are individualized to be performed by key clinicians in the surgical pathway. Each checklist is to be used as a last point of check before transfer to the next segment of the pathway, ensuring good planning and compliance with existing perioperative care protocols at all transfer points. Figure 2 displays the combined SURPASS and WHO SSC checklists across the surgical pathway as implemented in this study.

Before the trial, validation of the SURPASS checklist content into a Norwegian context was performed in a neurosurgical setting.^22^ Before checklist implementation in a new department, tailoring for specific conditions in the different Norwegian departments was performed in accordance with advice in the WHO implementation manual.^21^ Implementation of SURPASS was informed by our team’s extensive experience with implementing the WHO SSC in Norway^23^ and also by recently compiled implementation strategies for health care compendium developed by implementation scientists.^24^ In brief, the implementation strategy included educational sessions with frontline clinicians emphasizing why the checklists should be used, their evidence, and the practicalities of how to apply them.^25,26^ Individual coaching was offered by the research team. Moreover, an information campaign in the trial departments was performed through distribution of printed posters and emails to staff. Service managers and key clinicians in the different departments were designated champions of the SURPASS intervention. Last, audit and feedback on SURPASS implementation fidelity (ie, quality of application) was provided through regular compliance reports sent directly to all service managers by the research team.

### Outcome Measures

Primary outcomes were in-hospital morbidity (complications, emergency reoperations, and 30-day readmissions) and all-cause 30-day postoperative mortality. The secondary outcome measure was LOS.

### Inclusion and Exclusion Criteria

The study included in-hospital patients of all ages undergoing an elective or an emergency surgical procedure. Excluded were radiological interventions, donor surgery, extracorporeal membrane oxygenation procedures, outpatients, and patients who declined to consent to the study.

### Data Collection and Handling

Complications were investigated according to the *International Statistical Classification of Diseases and Related Health Problems, Tenth Revision* (*ICD-10*) as routinely recorded by physicians. The method for validating the reported in-hospital complications has been described previously.^27^ For the present study, all 155 *ICD-10* complication codes included in the analyses were verified against each patient’s medical records by the research team.

Data on mortality, LOS, patient characteristics, and surgical procedures were collected from the hospitals’ electronic administrative systems and verified against each patient’s medical record. Checklist data were combined with outcome data after this verification procedure. Patient characteristics included age, sex, American Society of Anesthesiologists (ASA) physical health classification, urgency of surgery, type of surgery, type of anesthesia, and time (month and year) of operation. Checklist adherence (ie, fidelity of application) was recorded per SURPASS checklist item and as the proportion of individual checklists with all items checked. Thus, for the preoperative SURPASS, the proportions were 0, 0.20 for 1 checklist, 0.40 for 2, 0.60 for 3, 0.80 for 4, and 1.00 for 5 (because these have 5 parts in all). For the postoperative SURPASS and WHO SSC, the proportions were 0, 0.33 for 1 checklist, 0.66 for 2, and 1.00 for 3 (because these have 3 parts in all). All data were collected as part of daily routine patient documentation, with staff blinded to outcome measures. All data handlers were blinded to checklists used in the care of individual patients.

### Statistical Analysis

Data were analyzed from September 25, 2018, to March 29, 2019. Characteristics of the preoperative and postintervention procedures and patient data were compared using the Pearson exact test with Bonferroni corrections for categorical variables and Gosset *t* tests for continuous variables. Intention-to-treat analyses were performed to evaluate changes in complication rates with comparison before and after the intervention regardless of SURPASS compliance. Multiple logistic regression analysis was used to evaluate associations of SURPASS with patient outcomes and including actual adherence to the checklists. Multiple Cox proportional hazards regression was used to analyze LOS. The individual checklists included in the analyses had all items checked. Preintervention and postintervention stages were analyzed while adjusting for time associations and other possible confounders in the logistic regression model,^18,28^ including age, sex, urgency of operation, ASA classification, anesthesia given, surgical specialty, point of time for inclusion in the study, and WHO SSC and SURPASS checklist adherence. Intention-to-treat analyses were adjusted for the same variables, except SURPASS adherence (as a proportion, as described above). With an expected mortality rate decrease from 0.015 to 0.008, a sample size of 3641 patients undergoing surgery in both preintervention and postintervention groups was required to achieve a power of 80% with an α value set to .05 (2 tailed). Intracluster correlation was not considered to affect the study power owing to heterogeneity within and between departments. The results for complications and mortality are reported as odds ratios (ORs) with 95% CIs and for LOS as hazard ratios (HRs). Two-sided *P* ≤ .05 was set as statistically significant. Power calculations were performed with SPPS Sample Power 2. Statistical analyses were performed using SPSS, version 24 (IBM Corporation).

## Results

The study included 3892 procedures at baseline and 5117 procedures in the intervention periods during the 29 months, from November 1, 2012, to March 31, 2015 (Figure 1). A total of 7772 unique patients underwent 9009 procedures (mean [SD] patient age, 51.7 [22.2] years) in 8515 admissions within the study. Characteristics of patients and surgical procedures are reported in Table 1. The inclusion of gynecology as one of the study departments contributed to an overall higher proportion of women (5601 women [62.2%] and 3408 men [37.8%]; *P* < .001). A total of 5117 intervention procedures (mean [SD] patient age, 51.8 [22.4] years; 2913 women [56.9%] and 2204 men [43.1%]) and 3892 controls (mean [SD] patient age, 51.5 [21.8] years; 2688 women [69.1%] and 1204 men [30.9%]) were included.

**Table 1.** Characteristics of 9009 Surgical Procedures in a Stepped-Wedge Cluster Nonrandomized Clinical Trial

Characteristic	Study group^a^		*P* value^d^
	Control (n = 3892)^b^	Intervention (n = 5117)^c^	
Male sex	1204 (30.9)	2204 (43.1)	<.001
Age, mean (SD), y	51.5 (21.8)	51.8 (22.4)	.49
ASA risk score^e^			
I	1020 (26.2)	1385 (27.1)	.14
II	2115 (54.4)	2630 (51.4)	
III	706 (18.2)	998 (19.5)	
IV	44 (1.1)	100 (2.0)	
V	1 (0.02)	3 (0.1)	
Surgery			
Elective	1878 (48.3)	2270 (44.4)	<.001
Emergency	2014 (51.7)	2847 (55.6)	
Anesthesia			
Regional	1310 (33.7)	1794 (35.1)	.172
General	2582 (66.3)	3323 (64.9)	
Surgical specialty			
Neurosurgery	636 (16.3)	1903 (37.2)	<.001
Orthopedics	1827 (46.9)	2612 (51.0)	
Gynecology	1429 (36.7)	602 (11.8)	
SURPASS preoperative checklists, No.^f^			
0	NA	216 (4.2)	NA
1	NA	503 (9.8)	
2	NA	1034 (20.2)	
3	NA	1903 (37.2)	
4	NA	1176 (23.0)	
5	NA	285 (5.6)	
WHO SSC intraoperative checklists, No.^f^			
0	48 (1.2)	39 (0.8)	<.001
1	192 (4.9)	251 (4.9)	
2	808 (20.8)	1442 (28.2)	
3	2844 (73.1)	3385 (66.2)	
SURPASS postoperative checklists, No.^f^			
0	NA	1397 (27.3)	NA
1	NA	2789 (54.5)	
2	NA	595 (11.6)	
3	NA	336 (6.6)	

Abbreviations: ASA, American Society of Anesthesiologists’ risk score; NA, not applicable; SURPASS, Surgical Patient Safety System; WHO SSC, World Health Organization surgical safety checklist.

^a^ Unless otherwise indicated, data are expressed as number (percentage) of procedures. Percentages have been rounded and may not total 100. Data are from 1 hospital in Western Norway from November 2012 through March 2015.

^b^ Includes 3274 unique patients.

^c^ Includes 4498 unique patients.

^d^ Calculated from Pearson exact test with Bonferroni corrections except ASA risk score (not exact test) and age (Gosset *t* test).

^e^ Missing for 6 control group procedures and 1 intervention group procedure. Higher scores indicate more comorbidities.

^f^ All items of individual checklists checked.

In total, 1418 of 9009 procedures (15.7%) were associated with 1 or more complications (Table 2). In adjusted intention-to-treat analyses, the number of complications decreased (OR, 0.73; 95% CI, 0.54-0.98; *P* = .04).

**Table 2.** Characteristics of Outcomes Before and After Intervention With SURPASS Checklists Added to WHO SSC

Outcome	Study group^a^		*P* value^b^
	Control (n = 3892)	Intervention (n = 5117)	
Respiratory	41 (1.1)	76 (1.5)	.08
Pneumonia	34 (0.9)	69 (1.3)	.045
Respiratory other	10 (0.3)	13 (0.3)	>.99
Cardiac	31 (0.8)	27 (0.5)	.14
Cardiac arrhythmia	7 (0.2)	14 (0.3)	.39
Congestive heart failure	14 (0.4)	9 (0.2)	.10
Cardiac other	13 (0.3)	7 (0.1)	.07
Infections	89 (2.3)	161 (3.1)	.01
Sepsis	7 (0.2)	10 (0.2)	>.99
Surgical site	13 (0.3)	7 (0.1)	.07
Urinary tract	68 (1.7)	138 (2.7)	.003
Infections other	4 (0.1)	11 (0.2)	.30
Surgical wound rupture	7 (0.2)	4 (0.1)	.23
Nervous system	11 (0.3)	18 (0.4)	.58
Delirium	6 (0.2)	12 (0.2)	.48
Cerebral infarction	5 (0.1)	7 (0.1)	>.99
Bleeding	105 (2.7)	201 (3.9)	.001
Embolism	12 (0.3)	8 (0.2)	.17
Nutrition	21 (0.5)	85 (1.7)	<.001
Malnutrition	7 (0.2)	56 (1.1)	<.001
Other disorders	14 (0.4)	44 (0.9)	.003
Anesthesia	6 (0.2)	4 (0.1)	.35
Mechanical implantation	4 (0.1)	3 (0.1)	.71
Fall	0	5 (0.1)	.07
Other	65 (1.7)	73 (1.4)	.39
Emergency reoperations	153 (3.9)	218 (4.3)	.45
Readmissions^c^	128 (3.5)	149 (3.1)	.32
Overall complications^d^	574 (14.7)	844 (16.5)	.03
Length of stay, d^c^			
Mean (SD)	5.8 (17.7)	5.6 (5.7)	.43
Median (IQR)	4.0 (2.0-7.0)	4.1 (2.2-6.9)	
Mortality within 30 d in-hospital^e^^,^^f^	23 (0.7)	28 (0.6)	.67
Mortality after discharge^f^	24 (0.7)	32 (0.7)	>.99

Abbreviations: IQR, interquartile range; SURPASS, Surgical Patient Safety System; WHO SSC, World Health Organization surgical safety checklist.

^a^ Unless otherwise indicated, data are expressed as number (percentage) of procedures.

^b^ Calculated using the 2-sided Pearson exact test with Bonferroni corrections for binary variables and Gosset *t* test for length of hospital stay.

^c^ Includes 3680 admissions in the control group and 4835 in the intervention group.

^d^ Included in overall complications are 155 *International Statistical Classification of Diseases and Related Health Problems, Tenth Revision* complication codes verified from unique surgical procedures, and emergency reoperations and 30-day readmissions.

^e^ Indicates 30 days or less from first operation on last hospital admission.

^f^ Includes 3274 patients in the control group and 4498 in the intervention group.

To analyze associations of complications per procedure with preoperative and postoperative SURPASS added to the WHO SSC, multiple logistic regression analyses were performed accounting for the level of adherence. When adherence to the preoperative SURPASS checklists was achieved, adjusted analysis demonstrated a decrease in in-hospital complications (OR, 0.70; 95% CI, 0.50-0.98; *P* = .04) and emergency reoperations (OR, 0.42; 95% CI, 0.23-0.76; *P* = .004) (Table 3). Furthermore, adherence to the 3 postoperative SURPASS checklists was associated with a reduction of unplanned 30-day readmissions (OR, 0.32; 95% CI, 0.16-0.64; *P* = .001) in adjusted analyses.

**Table 3.** Results From Logistic Regression of the Effects of Preoperative and Postoperative SURPASS Checklists Plus WHO SSC on 1 or More Complications in 9002 Surgical Procedures^a^

Variables	Unadjusted model		Fully adjusted model	
	OR (95% CI)	*P* value	OR (95% CI)	*P* value
SURPASS preoperative	0.97 (0.82-1.15)	.74	0.70 (0.50-0.98)	.04
SURPASS postoperative	1.02 (0.99-1.05)	.20	1.01 (0.97-1.05)	.65
WHO SSC	0.72 (0.55-0.94)	.02	0.90 (0.68-1.19)	.46
Male sex	0.92 (0.82-1.04)	.18	0.97 (0.85-1.11)	.67
Age, per 10 y	1.24 (1.20-1.27)	<.001	1.11 (1.08-1.15)	<.001
Month of operation^b^	1.14 (1.06-1.22)	<.001	1.23 (1.08-1.40)	.002
ASA risk score^c^	2.21 (2.04-2.39)	<.001	1.80 (1.65-1.97)	<.001
Urgency of surgery				
Elective	1 [Reference]	<.001	1 [Reference]	<.001
Emergency	2.32 (2.05-2.62)		2.34 (2.02-2.71)	
Anesthesia				
General	1 [Reference]	<.001	1 [Reference]	.99
Regional	1.58 (1.40-1.77)		1.00 (0.87-1.16)	
Surgical specialty				
Neurosurgery	1 [Reference]	.006	1 [Reference]	.02
Orthopedics	1.12 (0.98-1.28)		0.82 (0.69-0.96)	
Gynecology	0.89 (0.75-1.05)		1.03 (0.84-1.28)	

Abbreviations: ASA, American Society of Anesthesiologists; OR, odds ratio, effect size; SURPASS, Surgical Patient Safety System; WHO SSC, World Health Organization surgical safety checklist.

^a^ Calculated as proportions of checklists used. SURPASS included 5 preoperative checklists and 1 postoperative postanesthesia care unit nurse checklist; WHO SCC, 3 checklists. Preoperative SURPASS includes 0 for no checklist and 1 to 5 checklists (proportions, 0.20, 0.40, 0.60, 0.80, and 1.00); postoperative SURPASS and WHO SSC, 0 for no checklist and 1 to 3 checklists (proportions, 0.33, 0.66, and 1.00).

^b^ Time for inclusion in the study, per year.

^c^ Scores range from I to V, with higher scores indicating greater comorbidities.

Analyzing time trends in adjusted Cox proportional hazards regression showed an overall shorter LOS from early to later in the study period (ie, an increasing chance of earlier discharge; HR, 1.07 per year; 95% CI, 1.02-1.13; *P* = .003). Added use of the SURPASS checklists showed no significant associations with LOS.

The 30-day in-hospital mortality associated with using the preoperative SURPASS was nonsignificant (OR, 0.28; 95% CI, 0.04-1.78; *P* = .17). For postoperative SURPASS, the association was likewise nonsignificant (OR, 0.86; 95% CI, 0.68-1.08; *P* = .18). Similarly, there was no change in 30-day mortality after discharge associated with use of either the preoperative SURPASS (OR, 1.67; 95% CI, 0.38-7.44; *P* = .50) or the postoperative SURPASS (OR, 0.64; 95% CI, 0.17-2.45; *P* = .51) in all adjusted analyses.

The 3 parallel control surgical units included 9678 procedures during the study period (mean [SD] patient age, 57.4 [22.2] years; 4124 women [42.6%] and 5554 men [57.4%]). A CONSORT flow diagram describing eligible procedures is represented in the eFigure in Supplement 2. Characteristics of the procedures and outcome measures are reported in eTables 1 and 2 in Supplement 2, respectively. There was an overall decrease in LOS in the control units during the study period, with an increased chance of being more rapidly discharged (HR, 1.07 per year; 95% CI, 1.04-1.11; *P* < .001). We also found an increase in complications over time (OR, 1.09; 95% CI, 1.01-1.17; *P* = .04) (eTable 3 in Supplement 2). In adjusted analyses, no changes were observed in emergency reoperations (OR, 0.96; 95% CI, 0.82-1.12; *P* = .57), 30-day readmissions (OR, 1.17; 95% CI, 0.96-1.42; *P* = .11), 30-day in-hospital mortality (OR, 0.95; 95% CI, 0.70-1.29; *P* = .73), or 30-day mortality after discharge (OR, 1.14; 95% CI, 0.81-1.59; *P* = .46) in these departments.

## Discussion

Findings from this study demonstrate that adding the preoperative and postoperative SURPASS checklists to the intraoperative WHO SSC may be clinically advantageous. We found that the joint application of the 2 surgical checklist systems was associated with reduced in-hospital complications, emergency reoperations, and hospital readmissions.

A decade ago, the WHO SSC was initially implemented in 2 Norwegian hospitals (one being the present trial hospital), resulting in a 42% relative risk reduction of complications from 19.9% to 12.4%.^23^ Although the WHO SSC has become national clinical policy for surgery, evidence shows that surgical complications often originate outside the operating room.^12,13,14^ Logically, this outcome suggests that a checklist to improve flow of information and completeness of requisite clinical care protocols before a patient reaches the operating room can reduce unwanted variation in preparation and planning and improve care.^29^ Our findings suggest that effective application of the preoperative SURPASS may achieve this. The reduction in emergency reoperations when preoperative SURPASS had been used replicates studies showing a decrease in reoperations after implementing intraoperative surgical checklists.^20,30,31^

Furthermore, better use of the 3 postoperative SURPASS checklists was associated with decreased readmissions to hospital within 30 days. Improved communications optimize care delivery in transfer of patients to other units.^12,32,33,34,35^ The clinical associations we observed could be owing to the SURPASS discharge checklists supporting better preparation of patients when leaving the hospital (ie, plans for their medications and expectations regarding their ongoing recovery). Other studies have found that patient discharge is strengthened by use of checklists,^36^ and decreased readmissions have been linked to use of the WHO SSC.^37^

The parallel control units had increased complication rates over time, whereas rates of emergency reoperations, 30-day readmissions, and mortality remained unchanged. Over time, we observed an overall increased complication rate in both trial and control units. We do not have data directly addressing this finding. We hypothesize, however, that the national context of the study may account for this pattern. National economic incentive systems reimbursing *ICD-10* codes for complicated admissions have increased hospitals’ focus on coding practices.^38^ A possible explanation may be increased hospital focus on more accurate coding practice for reimbursement purposes by individual physicians throughout the study period. The increase in complications is unlikely to reflect a lack of effect from the checklist intervention, because when adjusted regression analyses were performed, the intervention was associated with lower risk of complications. Also, stricter adherence to the SURPASS checklists had a lower risk of complications than looser adherence, indicating a dose-response effect. Furthermore, use of the stepped-wedge design allowed us to adjust for time trends in complication rates.^17^ Both the trial departments and the control units had an overall decrease in LOS over time, and LOS was not associated with use of the SURPASS in the intervention departments. This finding contrasts with those of previous studies, which showed reduction in LOS with checklist use.^23,39^ We consider it possible that maximum reduction of LOS had been reached in our study owing to the national Norwegian context. Specifically, a national coordination reform took effect in January 2012.^40^ One of the main goals of the reform was to reduce LOS in hospitals by a build-up and enhancement of publicly funded nursing homes. This national policy program likely affected discharge decision-making throughout the study period and thus affected our findings. Our findings cannot directly support this explanation, which can be evaluated further through longitudinal outcome studies.

### Strengths and Limitations

This study has several strengths, including the prestudy SURPASS validation process, study design, long-term collection of data, and strong engagement from hospital leaders, managers, and influential clinicians when implementing the SURPASS intervention, thus achieving good fidelity. In addition, the validation procedures with exact and extensive verification of in-hospital *ICD-10* codes for complications, emergency reoperations, readmissions, mortality, and LOS from patient records linked to actual checklist adherence allowed reliable outcome measurement.^27^ Furthermore, the study allows distinguishing which checklists are associated with improvements on which outcomes. For example, combined use of the preoperative and postanesthesia care unit nurses’ SURPASS with the WHO SSC may improve in-hospital complications, in-hospital mortality, and LOS. Use of preoperative SURPASS checklists and the WHO SSC may influence emergency reoperations. A combined use of preoperative and postoperative SURPASS and WHO SSC may influence unplanned hospital readmissions and mortality after discharge.

The study also has limitations. The nonsignificant change in mortality could be owing to an underpowered sample size. The calculation was performed in 2012 based on the published literature.^15,20^ However, the number of patients dying in our sample was lower than anticipated. Furthermore, an important consideration is whether there could be any residual confounders explaining the observed higher rate of complications after the intervention. For example, were more complex procedures performed in sicker patients after the intervention? In Table 1, we showed that there is no difference in comorbidity (ASA classification) between control and intervention departments. In the regression analyses, we have adjusted for case mixes, including age, sex, emergency procedures, ASA classification, anesthesia given, surgical specialty, point of time for inclusion in the study, and checklist use. Additional comorbidity measures such as the Charlson comorbidity index were not part of the original study protocol. However, with these rigorous adjusted analyses, we believe that very little residual confounding has remained unexplained.

The parallel control units contributed different surgical procedures and specialties to the trial compared with the intervention departments. Comparing outcome data on similar procedures and specialties would have been ideal. However, morbidity and mortality trends in the parallel controls were similar to those of the intervention departments. The actual complexity of the SURPASS intervention, involving different professional groups across different departments, added an inherent limitation, because randomizing the start-up of the intervention with the time and resources available became unfeasible.

In addition, overall high-fidelity application of all checklists across all professional groups for all surgical procedures was not obtained. Known implementation barriers affect checklist use globally (eg, information technology systems, checklist and personnel flow, checklist resistance, and/or checklist fatigue) and could have resulted in underestimations of the sizes of associations of intervention and clinical outcomes in our analyses. Other investigators^41^ have also raised these issues. Further studies of how to improve fidelity in delivering clinically effective checklists in surgical pathways are warranted.

## Conclusions

Our findings suggest that combinations of the WHO and SURPASS checklists throughout the perioperative pathway may be clinically advantageous in improving processes of care and patient safety further with reductions in complications, reoperations, and readmissions beyond what sole use of the WHO checklist in the operating room achieves. The WHO checklist has been adopted globally for use in operating rooms. The next step to increase surgical patient safety is to use safety checklists throughout the perioperative pathway, as when combining the WHO checklist with SURPASS checklists. Rigorous large-scale multicenter randomized clinical trials are recommended to investigate this further.

## Notes

Supplement 1. Trial Protocol

Supplement 2. eFigure. CONSORT Flow Diagram eTable 1. Characteristics of 9678 Surgical Procedures With Care as Usual Over 29 Months in 3 Control Hospitals in Western Norway From November 1, 2012, Through March 31, 2015 eTable 2. Characteristics of Outcomes in 9678 Surgical Procedures Over 29 Months in 3 Control Hospitals in Western Norway From November 1, 2012, Through March 31, 2015 eTable 3. Results From Logistic Regression of Change in 1 or More Verified Complications in 9669 Surgical Procedures With Care as Usual in 3 Hospitals in Western Norway Over 29 Months, From November 1, 2012, Through March 31, 2015
