# Supplementary material for: Clinical Efficacy of Combined Surgical Patient Safety System and the World Health Organization’s Checklists in Surgery: A Nonrandomized Clinical Trial
Source: JAMA Surg. 2020 May 13;155(7):562–70. doi: 10.1001/jamasurg.2020.0989 (PMC7221852; doi:10.1001/jamasurg.2020.0989)
Supplement: Supplement 1. — Trial Protocol [file jamasurg-155-562-s001.pdf]

ClinicalTrials.gov Identifier: NCT01872195

PROTOCOL:

Effects of Checklists in Surgical Care - a Study on Complications, Death and Quality of Patient Administrative Data

Detailed Description:

1.0 Background

Surgical procedures are high risk events and patients may suffer complications or die post operatively. A report from an on-going patient safety campaign "In Safe Hands" lead by the Norwegian Knowledge Centre for the Health Services reveals that approximately 16 % of all Norwegian hospital admissions in 2010 involved an adverse event (AE) (Deilkås, 2011). A review study on AEs in 2008 included a wide range of in-hospital patients from Australia, Canada, New Zealand, the United Kingdom, and the United States of America (US) (de Vries et al., 2008). 9 % of the patients experienced an AE, with 7. 4 % of these ending fatally. The majority of the AEs occurred during surgical treatment or was related to drug administration. The authors claimed that almost half of these could have been prevented if checklists covering the entire surgical pathway had been used (de Vries et al., 2008). Implementation of such a system, called the Surgical Patient Safety System (SURPASS) did in fact result in a reduction of in-hospital morbidity (from 27.3% to 16.7%) and mortality (from 1.5 % to 0.8 %) (de Vries, 2010).

Patient safety checklists have been introduced and recommended as a standard of surgical care (Birkmeyer, 2010; de Vries et al., 2011). Studies based on data from electronic patient administrative systems show that checklist use may reduce mortality and morbidity in surgery (de Vries et al., 2010; van Klei et al., 2012; Haynes et al., 2009). Safe Surgery checklists have been recommended by the World Health Organization (WHO) since 2008 as a strategy to avoid adverse events (AE) during surgery. More than 6000 hospitals have implemented Safe Surgery checklists in their operating theatres (OTs) (http://www.who.int/patientsafety/safesurgery/en/), including Haukeland University Hospital (HUH).

This multicentre research project will also introduce a system of patient safety checklists at each point of care during the surgical patients' stay, not only in the operating theatres (OTs). The system combines new checklists on patient care (parts of SURPASS) with the already established Safe Surgery checklist (WHO) in the OTs. At the same time securing reliability, validity and quality of the patient, morbidity and mortality data will be an essential part of the study.

Today the discharging physician reviews the medical journal and makes a medical summary including coding diseases and complications relevant for the current admission. International Classification of Diseases (ICD-10) codes are used to set diagnoses for clinical, epidemiological and quality purposes (http://www.who.int/classifications/icd/ICD10Volume2_en_2010.pdf). The ICD-10 codes are also used for registrations on national mortality and morbidity in the Norwegian National Patient Register (NPR). Questions have been raised as to the accuracy and quality of the data in such registers in Norway, e.g. in patients with sepsis (Flaatten, 2004), and intensive care patients (Aardal et al., 2005). In a Danish

study on relations between ICD-10 coding in the National Registry of Patients and the hospitals' discharge summary and medical records, a high reliability between ICD-10 scores and co-morbidity was found (Thygesen et al., 2011). To our knowledge similar studies have not been done in Norway. As a crucial part of this investigation we concurrently will evaluate the reliability and validity of our patient administrative data by comparing the post discharge ICD-10 codes to actual data available directly from medical journal systems as documented by health care personnel in the journal texts.

2.0 Objective

The main objectives of this study are to:

- Perform a systematic review of published studies on effects of safety checklists in medicine.
- Explore effects on morbidity and mortality after implementing a system of patient safety checklists at each point of care during the surgical patients' stay (elements of SURPASS and the WHO Safe Surgery list combined), in a cohort of surgical patients in different surgical departments in one hospital, with patients from departments not having the system introduced serving as controls from three hospitals.
- Investigate the validity of the post discharge ICD-10 codes for complications compared to actual information found in medical journal systems texts.

    Discrepancies between patient information on complications registered as ICD-10 codes and information on complications documented in the actual electronic patient journal

    Registration of ICD-10 codes on complications and complications documented in the actual electronic patient journal will be registered separately and then compared as to discrepancies between these. This is done to evaluate and validate complication data (ICD-10 codes) used for primary outcome measures.

This project aims to produce a systematic review on present knowledge on effects of using safety checklists in medicine. Implementation of a checklist system throughout surgical care may reduce patient morbidity and mortality. The reliability of patient data is crucial to make firm conclusions as to such effects. This project aims to investigate if such morbidity and mortality effects are obtainable in two Norwegian hospitals while at the same time making a crucial evaluation of the patient data used in this study itself.

We hypothesise

1. An updated systematic review of the research literature provide evidence that safety checklists use does enhance safety and reduces patient mortality and morbidity
2. Implementation of the patient safety checklist system will reduce patient mortality and morbidity in the checklist cohort, and subsequent effects on length of stay
3. The sensitivity and specificity of ICD-10 coding vs. medical journal information is poor, with study results to be adjusted accordingly.

3.0 Methods 3.1 The projects and design

1. Systematic review.

   A systematic review on effects of safety checklists in medicine was done on May 29th, 2012 in the databases MEDLINE, Cochrane library, EMBASE and Web of Science, limited to only humans. The criteria were pre-set and included all time published quantitative studies in any language in the in-hospital and pre-hospital setting where safety checklists were the sole intervention, and effects of using checklists, generated as measurable outcomes. 7408 singular articles were found. To ensure the transparency of the reviewing process we used the PRISMA guidelines (Liberati et al., 2009). 34 studies met our inclusion criteria. The majority of the included studies measured effects pre-and post-intervention and was classified as having an observational design. . This systematic review has identified that safety checklists can be effective safety tools in various clinical settings. Their use has reduced patient mortality and morbidity. In addition, safety checklist use has been associated with better human performance, improved compliance with evidence-based practices, promoted consistency of care, and reduction of technical omissions. None of the included studies reported that safety checklists have negative effects on patient safety issues.

2. Implement the new patient safety checklist system and measure effects on morbidity, mortality and length of hospital stay.

   A prospective stepped wedge trial design (Brown & Lilford, 2006; Brown et al., 2008) will be used when implementing the validated patient safety checklist system in the Neurosurgical Department, the Orthopaedic Clinic and the Department of Gynaecology and Obstetrics at HUH. Patients from departments not using the patient safety checklist system serve as controls, this includes the Head and Neck Clinic (HUH), the Thoracic Surgery Section of the Heart Department (HUH) and two hospitals outside our own municipality (Health Trust Førde, and Health Trust Fonna - Haugesund Hospital). Primary end-points to be measured prospectively include length of hospital stay and morbidity and mortality utilizing the ICD-10 codes for complications collected electronically from the hospital patient administrative systems.

3. Validation of morbidity and mortality data.

   Today ICD-10 codes are produced by discharging physicians to summarize diagnoses at discharge and any complications having occurred during patient stay. In order to validate HUH's and Health Trust Førde's ICD-10 coding on patient morbidity and mortality we will randomize inclusion for quality check comparing the ICD-10 codes used at discharge to all actual information on morbidity and mortality as documented in the electronic patient journal (EPJ) - DIPS. This validation should include approximately 700 patients, all having undergone major surgery. Such a comparison is essential to gain knowledge

on the quality of generated ICD-10 data and thus important to the quality of results in this study.

3.2 Intervention study sample

Three surgical units at HUH (Department of Neurosurgery, Orthopaedic Clinic, and Department of Gynaecology and Obstetrics) will have the checklist system implemented. Approximately 3700 patients will be included before and 3700 patients after checklist implementation. The Control Group includes 7400 patients.

3.4 Data collection

For the study on mortality and morbidity we will extract ICD-10 codes used at discharge from the hospitals NPR file, as all Norwegian hospitals report their ICD-10 codes and procedure codes to NPR. In addition to registering all ICD-10 codes on each patient, we will collect demographic data (age, gender, height and weight), American Society of Anaesthesiologists Physical Health Classification (ASA), dates of admission and discharge, and all surgical procedures and major treatments. Data will be processed through Webport using a system previously developed locally for the WHO Surgical Safety Checklist project.

The primary end points, morbidity and mortality, are registered during hospitalization and postoperatively up to 30 days. Morbidity will be registered as major complications according to the American College of Surgeons' National Surgical Quality Improvement Program (http://www.facs.org/cqi/outcomes.html): organ/space surgical site infection, wound dehiscence, deep vein thrombosis, pulmonary embolism, pneumonia, re-intubation, ventilator use longer than 24 hours, cardiac arrest, myocardial infarction, sepsis, shock, coma longer than 24 hours, prosthetic/graft failure, and bleeding. Additional complications to these, as reported by de Vries (2010) will be included in order to make comparisons possible.

The study investigating reliability and validity of the ICD-10 codes will be done in detail: A prospective random selection of 700 patients, 200 patients from Health Trust Førde and 500 patients from the HUH, all having undergone major surgery. Present knowledge should suggest one or several major complications caused by procedures or iatrogenic causes in at least 17 % the surgical patients (de Vries, 2010). Then an inclusion of 700 patients is needed in order to find such complications in 119 cases. We will identify all post discharge ICD-10 codes for each patient. These codes will be thoroughly reviewed for accuracy and completeness by comparing to the actual information as documented by physicians and nurses in the EPJs throughout the total hospital stay. Primary outcome is here to investigate that registered ICD-10 codes have adequate sensitivity and specificity compared to the information in the patients' medical journal.

3.5 Statistics

Descriptive and inferential statistical methods will be used to analyse data. Confidence intervals (95% CI) for sensitivity and specificity will be calculated using the normal approximation for the standard error of proportions.

Mortality and morbidity will be analysed as to time of measurement, e.g. pre and post intervention, and surgical unit, i.e. using or not using the checklist. Multiple regression analysis and other appropriate statistical tools will be used to adjust for covariates to mortality and morbidity. Calculation of sample size and power, with an expected mortality rate decrease (0.015 vs. 0.008) requires a sample size of 3641 patients in both baseline and post intervention groups with an alpha (0.05, 2-tailed), power is 80%. To calculate sample size and power for morbidity mitigation from 27% to 17% (de Vries et al., 2010) requires a much smaller sample size of 234 in baseline and post intervention groups to constitute an 80% power with alpha at 0.05, 2-tailed. Statistical analysis will be conducted with appropriate statistical software e.g. Statistical Package for the Social Sciences, Stata or R.

Study Type :	Interventional (Clinical Trial)
Actual Enrollment :	21000 participants
Allocation:	Non-Randomized
Intervention Model:	Parallel Assignment
Masking:	None (Open Label)
Primary Purpose:	Prevention
Official Title:	Effects of Checklists in Surgical Care - a Study on Morbidity, Mortality and Data Quality
Study Start Date :	June 2013
Actual Primary Completion Date :	March 2015
Actual Study Completion Date :	March 2015

Arms and Interventions

Go to

Top of PageStudy DescriptionStudy DesignArms and InterventionsOutcome MeasuresEligibility CriteriaContacts and LocationsMore Information

Arm	Intervention/treatment
Experimental: * Before checklists The comprehensive patient safety checklist system	Other: The comprehensive patient safety checklist system The comprehensive patient safety checklist system follows each patient from admission to discharge with separate short checklists at each point of care: On admission to the hospital and ward (operating theatre nurse, ward doctor, surgeon, anaesthesiologist, ward nurse - 5 lists), in the operating theatre (here covered by the WHO-Safe Surgery checklist),

	at the recovery/ICU unit (nurse- 1 list), at discharge from the hospital (ward doctor, ward nurse - 2 lists).
Experimental: * After checklists<br>Without the comprehensive patient safety checklist system	Other: The comprehensive patient safety checklist system<br>The comprehensive patient safety checklist system follows each patient from admission to discharge with separate short checklists at each point of care: On admission to the hospital and ward (operating theatre nurse, ward doctor, surgeon, anaesthesiologist, ward nurse - 5 lists), in the operating theatre (here covered by the WHO-Safe Surgery checklist), at the recovery/ICU unit (nurse- 1 list), at discharge from the hospital (ward doctor, ward nurse - 2 lists).

Outcome Measures

Primary Outcome Measures :

1. Number of patients with complications or death, as a measure of checklist use [Time Frame: One year]

   Register number of patients with defined complications or peri- or postoperative death before and after checklist implementation.

Secondary Outcome Measures :

1. Discrepancies between patient information on complications registered as ICD-10 codes and information on complications documented in the actual electronic patient journal [Time Frame: One year]

   Registration of ICD-10 codes on complications and complications documented in the actual electronic patient journal will be registered separately and then compared as to discrepancies between these. This is done to evaluate and validate complication data (ICD-10 codes) used for primary outcome measures.

Other Outcome Measures:

1. Length of hospital stay (days) as a measure of checklist use. [Time Frame: One year]

   Length of hospital stay will be measured both before and after checklist use to evaluate if such use may have effects on hospital stay.

Eligibility Criteria

Ages Eligible for Study:	Child, Adult, Older Adult
Sexes Eligible for Study:	All
Accepts Healthy Volunteers:	No

Criteria

Inclusion Criteria:

- All patients undergoing a surgical procedure from the Orthopaedic Clinic, the Department of Gynaecology and Obstetrics and the Neurosurgical Department at Haukeland University Hospital.

Exclusion Criteria:

- Radiology surgical interventions, donor surgery, out-patients and all patients who have made a written statement as to reservation to participate (use of patient data), and those who do not understand Norwegian spoken and written language will be excluded from data collection.

Locations

Norway

Haukeland University Hospital
Bergen, Norway, 5021

Sponsors and Collaborators

Haukeland University Hospital

Investigators

Principal Investigator: Eirik Søfteland, MD, PhD Haukeland University Hospital

Responsible Party:	Haukeland University Hospital
ClinicalTrials.gov Identifier:	NCT01872195 History of Changes
Other Study ID Numbers:	REK Vest 2012/560<br>Regional Health Authority (Other Grant/Funding Number: 911755)
First Posted:	June 7, 2013 Key Record Dates
Last Update Posted:	June 11, 2015
Last Verified:	June 2015

Keywords provided by Haukeland University Hospital:

Checklist
In-Hospital Mortality
In-Hospital Morbidity

Supplemental Table Amendments made to the protocol regarding the checklist-intervention study:

Original	Amendment	Time	Explanation
Study Type: Observational (Patient Registry) Primary Purpose: Other Interventional Study Model: Allocation: N/A	Study Type: Interventional Primary Purpose: Prevention Interventional Study Model: Parallel Assignment Allocation: Non-Randomized	September 24, 2013	Precision from original protocol-text
Exclusion criteria: When time to complete the checklist is insufficient when emergency patients need urgent assistance.	Radiology surgical interventions, donor surgery, outpatients, and those who do not understand Norwegian spoken and written language.	March 19, 2014	Precision
Patients from departments not using the patient safety checklist system serve as controls, this includes the Head and Neck Clinic (HUH), the Thoracic Surgery Section of Heart Department (HUH) and two hospitals outside our own municipality (Health Trust Førde and Health Trust Fonna – Haugesund hospital).	*The Head and Neck Clinic (HUH) declined to participate<br><br>*The Control group includes 7400 patients	May 11, 2015	*Specifying the number of patients included from Control hospitals.
Enrollment: 7400 (Anticipated)	15000 (Actual)	May 11, 2015	*Actual numbers of procedures included was not yet quality checked, but recruiting was finished.
Enrolment: 7400 (Anticipated)	21000 (Actual)	June 10, 2015	*Actual numbers of procedures

			included was not yet completely quality checked, but recruiting was finished.

4
