# Supplementary material for: Clinical Efficacy of Combined Surgical Patient Safety System and the World Health Organization’s Checklists in Surgery: A Nonrandomized Clinical Trial
Source: JAMA Surg. 2020 May 13;155(7):562–70. doi: 10.1001/jamasurg.2020.0989 (PMC7221852; doi:10.1001/jamasurg.2020.0989)
Supplement: Supplement 2. — eFigure. CONSORT Flow Diagram eTable 1. Characteristics of 9678 Surgical Procedures With Care as Usual Over 29 Months in 3 Control Hospitals in Western Norway From November 1, 2012, Through March 31, 2015 eTable 2. Characteristics of Outcomes in 9678 Surgical Procedures Over 29 Months in 3 Control Hospitals in Western Norway From November 1, 2012, Through March 31, 2015 eTable 3. Results From Logistic Regression of Change in 1 or More Verified Complications in 9669 Surgical Procedures With Care as Usual in 3 Hospitals in Western Norway Over 29 Months, From November 1, 2012, Through March 31, 2015 [file jamasurg-155-562-s002.pdf]

Supplementary Online Content

Storesund A, Haugen AS, Flaatten H, et al. Clinical efficacy of combined Surgical Patient Safety System and the World Health Organization's checklists in surgery: a nonrandomized clinical trial. *JAMA Surg*. Published online May 13, 2020. doi:10.1001/jamasurg.2020.0989

eFigure. CONSORT Flow Diagram

eTable 1. Characteristics of 9678 Surgical Procedures With Care as Usual Over 29 Months in 3 Control Hospitals in Western Norway From November 1, 2012, Through March 31, 2015

eTable 2. Characteristics of Outcomes in 9678 Surgical Procedures Over 29 Months in 3 Control Hospitals in Western Norway From November 1, 2012, Through March 31, 2015

eTable 3. Results From Logistic Regression of Change in 1 or More Verified Complications in 9669 Surgical Procedures With Care as Usual in 3 Hospitals in Western Norway Over 29 Months, From November 1, 2012, Through March 31, 2015

This supplementary material has been provided by the authors to give readers additional information about their work.

©2020 Storesund A et al. *JAMA Surgery*.

eFigure. CONSORT Flow Diagram

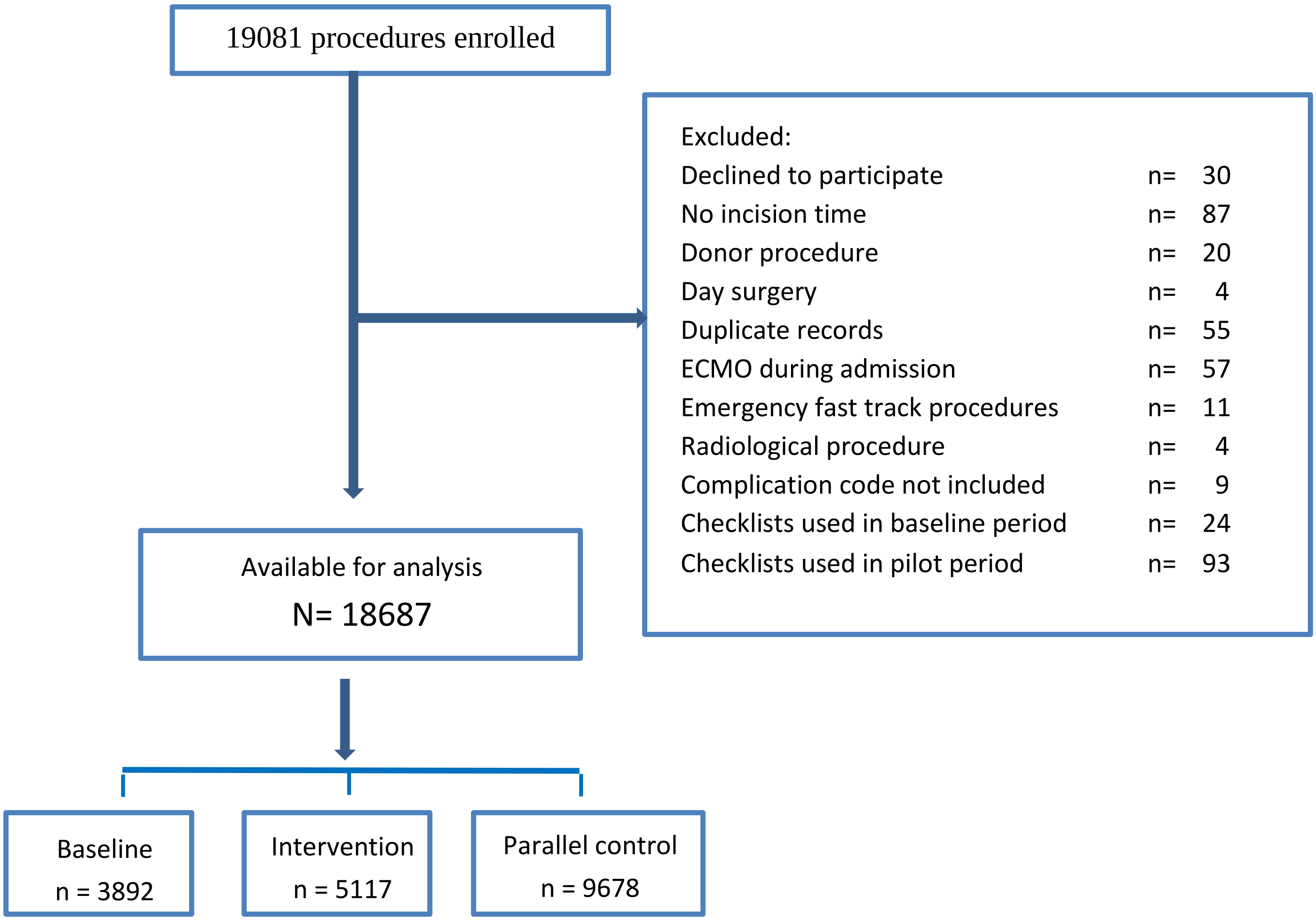

©2020 Storesund A et al. *JAMA Surgery*.

eTable 1. Characteristics of 9678 Surgical Procedures With Care as Usual Over 29 Months in 3 Control Hospitals in Western Norway From November 2012 Through March 2015

**Characteristic**	
Male sex, *n (%)*	5554 (57.4)
Age (years), *mean (SD)*	57.4 (22.2)
ASA [b)], *n (%)*	
I	1681 (17.4)
II	4294 (44.4)
III	3330 (34.4)
IV	356 (3.7)
V	8 (0.1)
Surgery, *n (%)*	
Elective	6146 (63.5)
Emergency	3532 (36.5)
Anesthesia, *n (%)*	
Regional	2510 (25.9)
General	7168 (74.1)
Surgical specialty, *n (%)*	
Thoracic surgery [c)]	2439 (25.2)
Central hospital I [d)]	5863 (60.6)
Central hospital II [e)]	1376 (14.2)

Abbreviations: ASA = American Society of Anesthesiologists' risk score; SD = standard deviation;
a) Unique patients = 8121; b) 9 missing ASA scores. c) Tertiary hospital; d) (Fonna) General surgery, Vascular surgery, Orthopedics, Ear/Nose/Throat surgery, Urology; e) (Førde) General surgery, Vascular surgery, Urology, Gastrointestinal surgery.

©2020 Storesund A et al. *JAMA Surgery*.

eTable 2. Characteristics of Outcomes in 9678 Surgical Procedures Over 29 Months in 3 Control Hospitals in Western Norway From November 2012 Through March 2015

**Outcome**	
	**n (%)**
Respiratory	453 (4.7)
Pneumonia	279 (2.9)
Respiratory other	222 (2.3)
Cardiac	570 (5.9)
Cardiac arrhythmia	492 (5.1)
Congestive heart failure	46 (0.5)
Cardiac other	48 (0.5)
Infections	307 (3.2)
Sepsis	31 (0.3)
Surgical site	89 (0.9)
Urinary tract	175 (1.8)
Infections other	19 (0.2)
Surgical wound rupture	30 (0.3)
Nervous system	50 (0.5)
Delirium	23 (0.2)
Cerebral infarction	29 (0.3)
Bleeding	310 (3.2)
Embolism	26 (0.3)
Nutrition	114 (1.2)
Malnutrition	24 (0.2)
Other disorders	94 (1.0)
Anaesthesia	7 (0.1)
Mechanical implantation	16 (0.2)
Fall	0 (0.0)
Other	155 (1.6)
Emergency reoperations	319 (3.3)
Readmission [a)]	212 (2.3)
Overall complications [b)]	1993 (20.6)
Length of stay in days, *mean (SD)/median (IQR)* [a)]	*6.5 (8.2)/ 4.3 (2.1 to 8.1)*
Mortality 30 days [c)] in-hospital	88 (1.1)
after discharge	73 (0.9)

Abbreviations: SD = standard deviation; IQR = interquartile range. a) n = 9027 admissions; b) Included in overall complications are 155 ICD-10 complication codes verified from unique surgical procedures, emergency reoperations and 30-day readmissions; c) 30 days or less from first operation on last hospital admission. n = 8121 patients.

©2020 Storesund A et al. *JAMA Surgery*.

eTable 3. Results From Logistic Regression of Change in 1 or More Verified Complications in 9669 Surgical Procedures With Care as Usual in 3 Hospitals in Western Norway Over 29 Months, From November 2012 Through March 2015

Variables	Unadjusted models			Fully adjusted model		
	**OR**	**95% CI**	**P-value**	**OR**	**95% CI**	**P- value**
Male sex	1.21	(1.09, 1.34)	< 0.001	0.98	(0.88, 1.10)	0.729
Age [a)]	1.31	(1.27, 1.35)	< 0.001	1.27	(1.22, 1.32)	< 0.001
Month for operation [b)]	1.08	(1.01, 1.16)	0.027	1.09	(1.01, 1.17)	0.037
ASA	2.98	(2.77, 3.21)	< 0.001	1.62	(1.47, 1.77)	< 0.001
Urgency of surgery			0.110			< 0.001
Elective	1.00	reference		1.00	reference	
Emergency	1.09	(0.98, 1.20)		1.75	(1.55, 1.97)	
Anesthesia			< 0.001			< 0.001
General	1.00	reference		1.00	reference	
Regional	0.54	(0.48, 0.62)		0.63	(0.54, 0.74)	
Surgical specialty			< 0.001			< 0.001
Thoracic surgery [c)]	1.00	reference		1.00	reference	
Central hospital I [d)]	0.20	(0.18, 0.22)		0.27	(0.23, 0.31)	
Central hospital II [e)]	0.19	(0.16, 0.22)		0.24	(0.20, 0.29)	

Abbreviations: OR = Odds Ratio, effect size; CI = Confidence interval; P = p-value from likelihood ratio test; no adjustment for multiplicity; ASA = American Society of Anesthesiologists' risk score, scale: 1-5.
a) Per 10 years; b) Time point for inclusion in the study, per year; c) Tertiary hospital; d) (Fonna) General surgery, Vascular surgery, Orthopedics, Ear/Nose/Throat surgery, Urology; e) (Førde) General surgery, Vascular surgery, Urology, Gastrointestinal surgery.

©2020 Storesund A et al. *JAMA Surgery*.
